# Biodegradable stents for biliary strictures after pediatric liver transplantation: a multicenter retrospective study

**DOI:** 10.1007/s00330-025-11930-5

**Published:** 2025-09-03

**Authors:** Paolo Marra, Daniel Barnes-Navarro, Lucía Fernández-Rodríguez, Giulio Barbiero, Simon Prosser McGuirk, Carla Gonzalez-Junyent, Iratxe Díez-Miranda, Jesus Quintero-Bernabeu, Teresa Hernández-Cabrero, Joan Novo-Torres, Maria Dolores Ponce-Dorrego, Jimena Gonzalez-Nieto, Michele Battistel, Maria Carla Minà, Stefano Groff, Conor J. A. Aleman, Rebecca Lucy Warren, Khalid Sharif, Chiara Ceriani, Riccardo Muglia, Lorenzo D’Antiga, Sandro Sironi, Mercedes Perez-Lafuente

**Affiliations:** 1 School of Medicine and Surgery, University of Milano-Bicocca, Milan, Italy; 2 Department of Radiology, ASST Papa Giovanni XXIII Hospital, Bergamo, Italy; 3 Interventional Radiology Unit, Hospital Universitario Vall d’Hebron, Barcelona, Spain; 4 Interventional Radiology Unit, Hospital Universitario La Paz, Madrid, Spain; 5 Radiology Unit, University Hospital of Padua, Padova, Italy; 6 Department of Radiology, Birmingham Women’s and Children’s Hospitals NHS Foundation Trust, Birmingham, UK; 7 Pediatric Hepatology and Liver Transplant Unit, Department of Pediatrics, Vall d’Hebron Hospital Campus, ERN Rare Liver ERN TransplantChild, Barcelona, Spain; 8 Institute of Radiology, University Hospital of Padua, Padova, Italy; 9 The Liver Unit, Birmingham Women’s and Children’s Hospitals NHS Foundation Trust, Birmingham, UK; 10 Paediatric Hepatology Gastroenterology and Transplantation, ASST Papa Giovanni XXIII Hospital, Bergamo, Italy

**Keywords:** Liver transplantation, Pediatrics, Cholangiography, Stents, Polydioxanone

## Abstract

**Objectives:**

Biodegradable biliary stents are used to treat benign biliary strictures in adults. However, there is limited data regarding their use in pediatric patients. This study aims to assess the efficacy and safety of biodegradable biliary stents following pediatric liver transplantation (pLT).

**Materials and methods:**

Consecutive pLT patients with benign biliary strictures were retrospectively evaluated in five tertiary centers between October 2014 and May 2024. All patients underwent percutaneous bilioplasty followed by the placement of self-expanding polydioxanone-based stents. Stricture features and treatment timing were assessed, as well as freedom from stricture recurrence and complications.

**Results:**

A total of 102 patients (52 males, 50 females; median age at treatment = 5 years, interquartile range (IQR) = 2–11 years) were included. At baseline, 58/102 (57%) had a stricture length ≥ 10 mm, and 53/102 (52%) had intrahepatic duct involvement. Stenting was performed a median of 55 days (IQR = 15–128 days) following biliary drainage. Technically successful stent placement was achieved in 101/102 (99%) cases, and low-grade complications occurred in 19/102 (19%). During a median follow-up of 793 days (IQR = 341–1529 days), 24/102 (24%) patients had stricture recurrence with an estimated median time to recurrence of 2915 days. Eight patients were lost to follow-up before recurrence. Logistic regression did not identify any factors that were independent predictors of stricture recurrence, while Cox regression showed that recoil/residual stenosis was independently associated with earlier recurrence (*p* = 0.005; HR = 5.334; 95% CI = 1.674–17).

**Conclusion:**

Biodegradable stents appear safe and effective for treating biliary strictures after pLT. Certain factors may contribute to failure and should inform patient selection and the optimal timing for stenting.

**Key Points:**

***Question*** *The management of biliary strictures after pediatric liver transplantation (pLT) poses several challenges due to the frailty of the population and refractoriness of the condition*.

***Findings*** *In this multicenter study self-expanding biodegradable biliary stents made of polydioxanone proved to be safe and effective for the treatment of biliary strictures after pLT*.

***Clinical relevance*** *Biodegradable biliary stents may improve the management of biliary strictures after pLT with a positive impact on outcomes and less invasiveness; some factors may help to predict the outcome and define the best timing for stenting*.

**Graphical Abstract:**

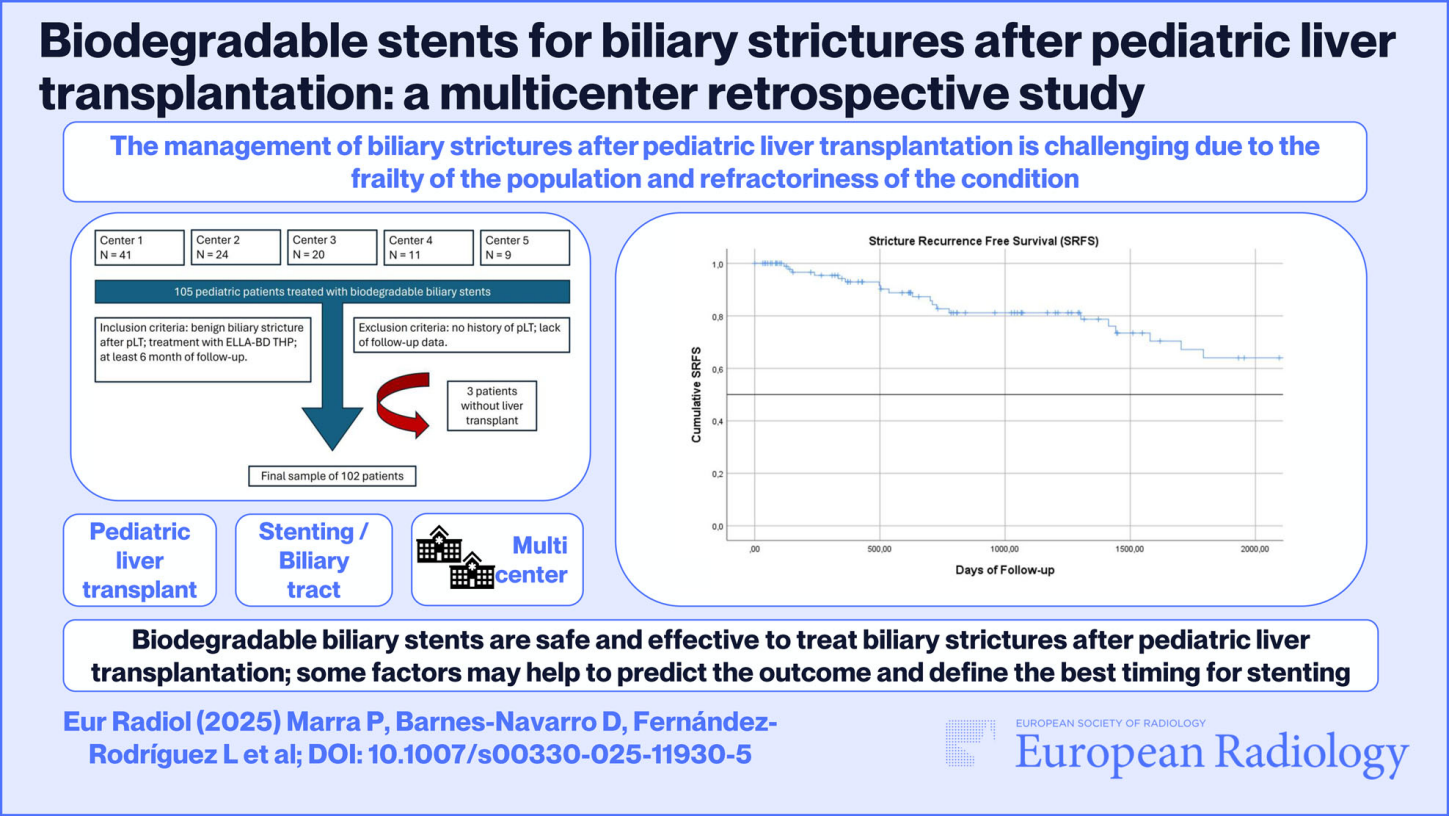

## Introduction

Biliary complications are relatively common after pediatric liver transplantation (pLT) [1, 2]. The treatment of biliary strictures may include percutaneous transhepatic cholangiography (PTC), endoscopic retrograde cholangiopancreatography, and/or surgical revision. The choice largely depends on the type of biliary reconstruction and the local expertise available [3–6]. Historically, the Roux-en-Y hepaticojejunostomy has been the biliary reconstruction procedure of choice in pediatric transplantation due to the high prevalence of biliary atresia and technical challenges related to the small size and frailty of the ducts of recipients; duct-to-duct reconstruction is performed in a small proportion of cases [7, 8]. In patients with a Roux-en-Y hepaticojejunostomy, PTC with bilioplasty and biliary drainage is currently the preferred treatment option due to the technical limitations of the endoscopic approach. However, there is still no consensus among expert centers on the optimal PTC protocol for managing biliary strictures [9–12].

Success rates for PTC and transluminal bilioplasty with balloon catheters reach approximately 70%, and while follow-up periods differ across studies, stricture relapse is usually observed within 2 years [10, 13, 14]. However, this strategy leaves an internal-external biliary drain in situ for prolonged periods. This is a major drawback, increasing the risk of infections [15], prolonging hospitalization, and leading to higher readmission rates. All these issues are particularly pertinent in pediatric patients, significantly impacting their quality of life. Moreover, pLT patients experiencing recalcitrant biliary strictures may require multiple interventions with associated increased ionizing radiation exposure.

The invasiveness of percutaneous retrieval procedures limits the use of internal biliary stents in pediatric patients, while permanent metallic stents are considered for palliation. Biodegradable stents, such as self-expanding polydioxanone stents, represent an attractive additional therapeutic option for treating biliary strictures in the pediatric population. Biodegradable stents were introduced over a decade ago and have seen increasing application, particularly in adult patients with benign biliary strictures [16–18] or post-liver transplantation [19]. The global distribution of these devices was limited due to the absence of CE-mark or FDA clearance. However, several pediatric liver transplant centers across Europe gained experience with these devices due to the urgent need for effective treatment strategies for children experiencing persistent biliary strictures. While numerous studies have shown positive outcomes in adults [16, 19], there is limited data regarding the feasibility, indications, efficacy, and safety of biodegradable stenting in children [20].

This study involving the largest multicenter experience with biodegradable stents for biliary strictures after pLT aims to evaluate the efficacy and safety of the treatment and analyze factors that may influence treatment outcomes.

## Materials and methods

### Study design and population

This is a multicenter retrospective analysis of patients with biliary strictures developed after pLT, who were treated with the same polydioxanone-braided biodegradable biliary stent (ELLA-BD THP; Ella-CS Ltd.) from January 1, 2014, to May 31, 2024, in five European referral centers for pLT. The centers were enrolled through a spontaneous survey sent from the institution leading the research, with a specific dataset to collect. Data were aggregated, anonymized and centrally analyzed. The need for consent was waived for the retrospective nature of the study that was authorized (n.reg. 20/24) by the local Ethics Committee of the institution leading the research. Some of the study patients were included in previously published research [20].

The inclusion criteria were: diagnosis of benign biliary stricture after pLT; treatment with ELLA-BD THP; at least 6 months of follow-up.

The exclusion criteria were: no history of pLT; lack of follow-up data.

**Fig. 1:**
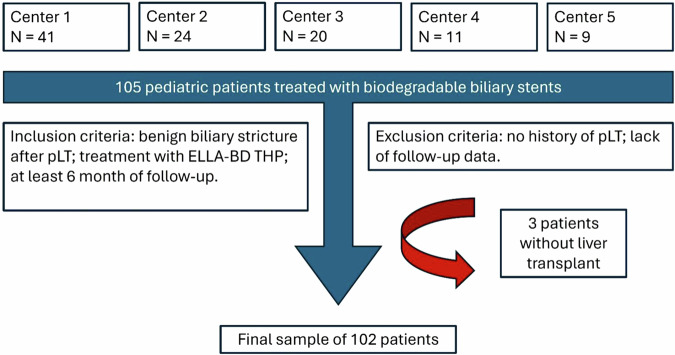
Flow diagram of the study; pLT, pediatric liver transplantation

From the multicenter survey, 105 patients were included in the aggregated dataset. Following exclusion criteria, 3 patients were removed to reach a final study population of 102 patients, as represented in Fig. 1.

The primary endpoint of the study was the freedom from stricture recurrence during follow-up. The secondary endpoints involved the feasibility of stent placement, safety in terms of complications and analysis of factors potentially affecting the outcomes.

### Treatment protocol, variables and outcome measures

The standard treatment approach for biliary strictures after pLT relied on PTC and bilioplasty in every center. There was variability among centers in terms of: time from the first PTC to ELLA-BD THP placement; number of bilioplasty sessions before biodegradable stent placement; employment and duration of internal-external biliary drainage before biodegradable stent placement; size of bilioplasty balloon and drainage catheter. The biliary strictures were classified based on cholangiography findings as: anastomotic or intrahepatic; < or ≥ 10 mm. All these variables were recorded and included in the multivariate analysis. The size of the ELLA stent ranged from 6 to 10 mm and was at least as large as the maximum balloon used for bilioplasty.

Previous biliary treatments, as well as features of the strictures including residual/recoil after bilioplasty, which were the main indications to biodegradable stenting in addition to poor tolerance of the internal-external drainage, were also considered in the multivariate analysis.

Due to the absence of a standardized protocol in the management of biliary strictures after pLT, every practitioner from the enrolling centers indicated the biodegradable stent implantation on a case-by-case analysis, upon multidisciplinary team discussion. During the enrollment period, ELLA-BD THP had no clearance from the FDA nor CE-Mark; therefore, each implantation was subject to an authorization request from the physicians to their respective regulatory agencies or Ethics Committee for custom-made use. A detailed informed consent form was signed by the patient’s parents or legal guardians.

All PTC procedures were conducted in the interventional angiographic suite according to each institution’s protocols, under monitored general anesthesia supplemented with local anesthesia. Prophylactic antibiotics were administered prior to all procedures. Sonographic guidance was employed to determine the optimal percutaneous transhepatic route for performing a peripheral biliary puncture while avoiding vascular structures. Biliary puncture and catheterization were executed using standard 22-gauge needles and 4-French coaxial introducers. Access for left lateral segment grafts was achieved via the left anterior subxiphoid approach, while right lateral segments and whole livers were accessed through the intercostal route. Standard 4-French catheters were used to catheterize the biliary tree, with strictures being crossed using 0.035-inch, 0.0018-inch, or 0.0014-inch hydrophilic guidewires. Strictures were then dilated using non-compliant high-pressure balloon catheters ranging from 4 mm to 12 mm in size. Intrahepatic strictures were dilated up to 6 mm, and anastomotic strictures were dilated up to 8 mm in infants and up to 12 mm in older children. Each dilation session lasted 3 min and was repeated thrice. Following bilioplasty, an internal-external biliary drain, ranging from 6-French to 12-French based on liver and biliary duct size, was typically inserted (except in cases of primary stenting). The drain was initially left for external gravity drainage for at least 24 h. If the patient exhibited no signs of fever or cholangitis, the catheter was subsequently clamped for internal drainage. In accordance with institutional protocols, post-bilioplasty, the internal-external biliary drainage tube was maintained in situ for a period ranging from 1 week to several months. During this time, one or more additional bilioplasty sessions were performed prior to stenting. Subsequent cholangiography was conducted, and the internal-external biliary drainage tube was replaced with a transhepatic 12-French introducer sheath. The biodegradable stent was loaded on an 11.8-French pull-back delivery system, which was advanced through the vascular sheath. For cases involving primary stenting, the delivery system was directly introduced on the guidewire without the use of an introducer sheath.

Bilioplasty was performed in preparation for stenting and could eventually be repeated after stent deployment to gain the nominal stent caliber. An intrastent catheter was occasionally left in place and removed within 30 days.

**Fig. 2:**
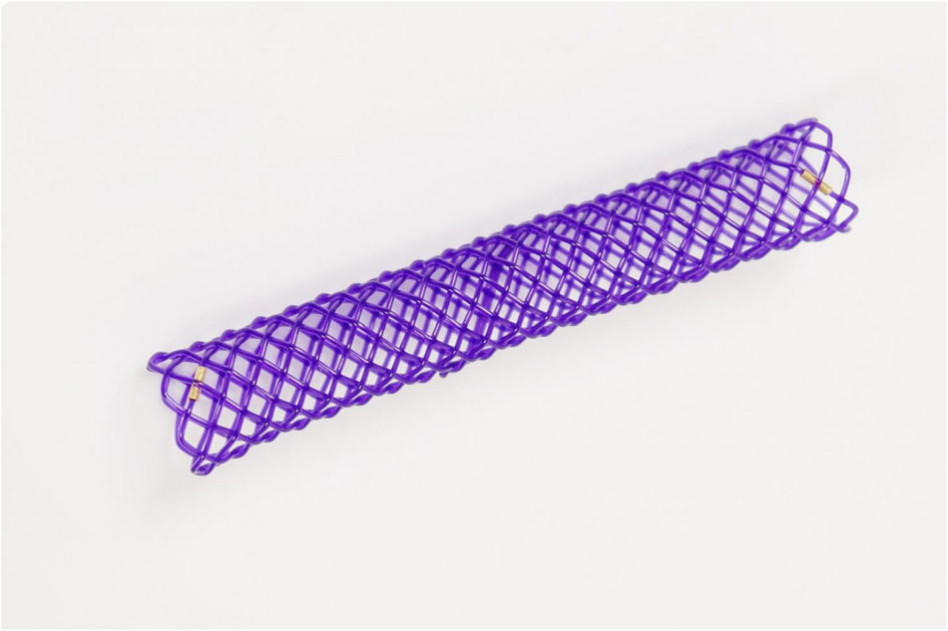
Macroscopic appearance of the self-expandable stent made of polydioxanone monofilament braided to form an uncovered mesh, with gold radiopaque markers at the extremities

Biodegradable stents were custom-made devices potentially available in a wide range of dimensions upon individual request. Standard sizes and lengths ranging from 6 to 10 mm and from 45 to 60 mm, respectively, were employed in the study according to the site of deployment and the maximum size of the bilioplasty catheter. The stent is made of a polydioxanone monofilament braided to form an uncovered mesh, with gold radiopaque markers at the extremities (Fig. 2).

All the patients adhered to a routine follow-up schedule, which varied according to institution protocols and on an individual basis. Typically, pLT patients were referred to regional or national centers for annual evaluations. Consequently, patients without any clinical updates by the year 2023 and with a follow-up period of less than 1 year were considered lost to follow-up. The treatment outcome was assessed through clinical visits, blood exams, including a liver panel for the screening of cholestasis and liver ultrasound. The event “stricture recurrence” was defined if clinical, laboratory or ultrasound signs of cholestasis were detected and the patient was censored accordingly. The follow-up time was recorded for each patient until censoring.

In addition to the above variables, many other factors were recorded and analyzed including: sex and age; indication to transplantation; putative etiology of biliary stricture; age at liver transplant; technical success of stent placement; complications (graded according to the CIRSE classification system) [21]; liver function tests including direct bilirubin, alkaline phosphatase (ALP) and gamma-glutamyl transferase (GGT) before treatment and before censoring.

### Statistical analysis

The data are presented as numbers with percentages for categorical variables and as medians and interquartile range (IQR) for continuous variables. Continuous variables were compared among categories using the Mann–Whitney test. Logistic regressions were employed to assess factors potentially influencing treatment outcome. Kaplan–Meier survival curves were built in function of the occurrence of the event “stricture recurrence” over the follow-up time, and Log Rank and Cox Regression analyses were performed to identify factors potentially affecting the time to recurrence. Odds Ratios (OR) and Hazard Ratios (HR) with 95% confidence intervals (95% CI) were reported if significant. All *p*-values were two-sided, and a significance level of 5% was established. Statistical analyses were conducted using SPSS (IBM, version 25).

## Results

### Baseline features

During the study period, a total of 729 PTCs were performed in 326 pLT patients across the five centers. The study population included 102 patients (52 males, 50 females; median age at treatment = 5 years, IQR = 2–11 years; median age at liver transplant = 1 year, IQR = 0.9–4.3 years). Baseline characteristics are detailed in Table 1. Briefly, 72/102 (71%) patients had already received a median of 2 (IQR = 1–3) previous treatments for biliary stricture after liver transplant. Several indications for liver transplant were recorded: the most commonly represented were biliary atresia and Alagille syndrome in 53/102 (52%); genetic or secondary cholestasis in 22/102 (22%); neoplasm in 12/102 (12%); a genetic metabolic disorder in 11/102 (11%); and liver failure in 4/102 (4%). The etiology of biliary stricture was unknown in 94/102 (93%) cases or supposed to be related to arterial obstruction in 4/102 (4%) or post-transplant bile leak in 3/102 (3%). The baseline laboratory values and the features of the strictures are detailed in Table 1. Briefly, the stricture involved only the surgical anastomosis in 49/102 (48%) cases, while in the remaining 53/102 (52%) cases it involved the intrahepatic ducts; the stricture length was ≥ 10 mm in 58/102 (57%) cases.

**Table 1:** Demographics and baseline clinical data

	Number	
Study population *N* = 102 patients		
Male	52	
Female	50	
Etiology of liver transplant		
Biliary Atresia/Alagille syndrome	53	
Genetic or secondary cholestasis	22	
Neoplasm	12	
Genetic metabolic disorder	11	
Liver failure	4	
Previous treatments for biliary stricture		
Yes	72	
No	30	
Baseline stricture features		
Only anastomotic	49	
Intrahepatic	53	
Presumed etiology of the stricture		
Unknown	95	
Ischemic	4	
Bile leak	3	
Stricture length		
≤ 10 mm	44	
> 10 mm	58	
	**Median**	**IQR**
Age at treatment (years)	5	1–11
Age at liver transplant (years)	1	0.9–4.3
Number of previous treatments	2	1–3
Baseline direct bilirubin (mg/dL)	0.44	0.23–1.74
Baseline alkaline phosphatase (UI/L)	275	101–599
Baseline gamma-glutamyl transferase (UI/L)	201	91–403

*IQR* interquartile range

### Treatment details

**Fig. 3:**
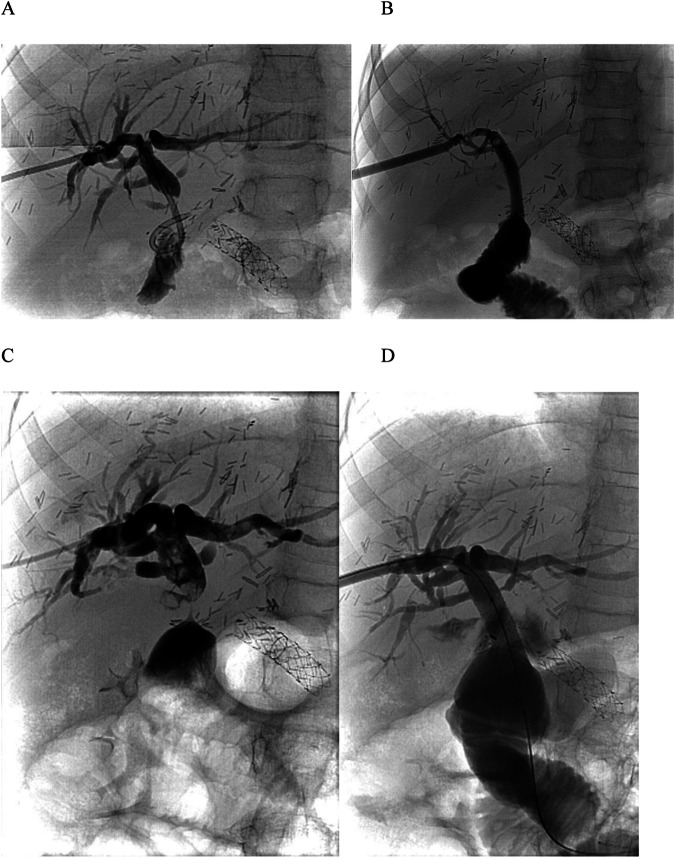
Anteroposterior percutaneous transhepatic cholangiography images of a recalcitrant anastomotic stricture in a 3-year-old boy after whole liver transplantation due to biliary atresia. **A** Image shows an external-internal biliary drainage placed after bilioplasty treatment of a hepaticojejunostomy stricture after whole liver transplantation; the metal stent was implanted to treat a portal vein stenosis. **B** Image acquired after placement of a transanastomotic 8 × 60 mm self-expanding biodegradable stent. **C** Image shows recurrence of the hepaticojejunostomy stricture 6 months after treatment. **D** Image shows intrastent bilioplasty performed with a 10 mm balloon after deployment of a new 10 × 60 mm self-expanding biodegradable stent. The kid was free from stricture recurrence during a 5-year follow-up

**Fig. 4:**
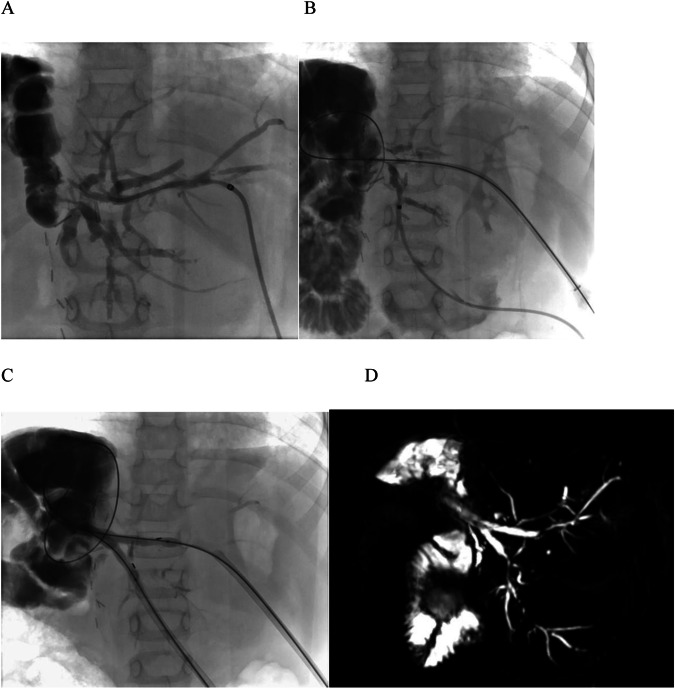
Treatment of complex anastomotic and intrahepatic strictures in a 9-year-old girl after split liver transplantation due to sclerosing cholangitis. **A** Anteroposterior cholangiography image shows access to segment 2 bile duct with multiple strictures involving the hepaticojejunostomy, the confluence between segmental ducts and intrahepatic ducts. **B** Anteroposterior cholangiography image acquired after cannulation of segment 3 bile duct. **C** Anteroposterior cholangiography image shows intrastent kissing balloon dilation performed after placement of two side-by-side self-expanding 8 × 45 mm biodegradable stents. **D** MR cholangiography image performed 3 weeks after stenting shows intrastent hyperintensity consistent with stent patency, with general improvement of bile duct dilation, despite persistence of some intrahepatic strictures

Treatment details are summarized in Table 2. Briefly, the biodegradable stents were implanted after a median of 55 days (IQR = 15–128 days) from the first PTC, while stent placement was performed the same day of the first PTC in 18 patients without maintenance of an internal-external biliary drainage. One biodegradable stent (Fig. 3) was used in 87/102 (85%) patients, while 2 stents (Fig. 4) were required in 15/102 (15%) patients. The patients received a median of 2 (IQR = 1–3) bilioplasty sessions with a median balloon size of 8 mm (IQR = 6–8 mm). Those who received an internal-external biliary drainage before biodegradable stent placement had catheters of a median size of 8 French (IQR = 8–10 French). Recoil/residual stenosis after bilioplasty was observed in 30/102 (29%) patients. Technical success of stent placement was achieved in 101/102 (99%) cases with a single case of intraprocedural migration. Complications occurred in 19/102 (19%) cases, including cholangitis, hemobilia and pancreatitis, all conservatively managed without sequelae (CIRSE grade 3).

**Table 2:** Procedure details

	Median	IQR
Time between the first percutaneous cholangiography and stent placement (days)	55	113
Number of bilioplasty sessions before stent placement	2	1–3
Maximum bilioplasty balloon size (mm)	8	6–8
Maximum drainage catheter size (French)	8	8–10
	**Number**	
Stent placement at the first percutaneous cholangiography		
Yes	18	
No	84	
Number of stents		
One	87	
Two	15	
Technical success of stent placement		
Yes	101	
No	1	
Recoil/residual stenosis		
Yes	30	
No	72	
Complications		
No	83	
Infectious	15	
Hemobilia	3	
Pancreatitis	1	
Clinical success		
Yes	78	
No	24	

*IQR* interquartile range

### Factors affecting treatment outcome

**Fig. 5:**
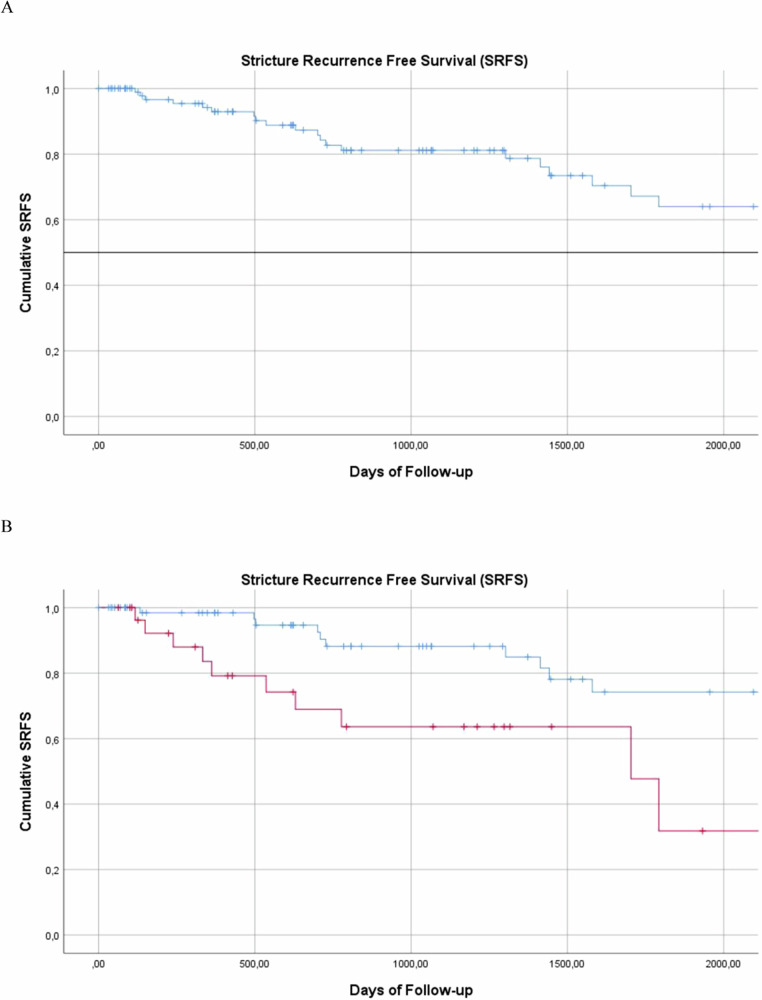
**A** The Kaplan–Meier curve shows survival without stricture recurrence (SRFS) in the study population. **B** The Kaplan–Meier curves display SRFS in subgroups based on the presence (red) or absence (blue) of recoil/residual stenosis after bilioplasty

During a median follow-up of 793 days (IQR = 341–1529 days), 24/102 (24%) patients had a stricture recurrence; of these, 13/102 (13%) were retreated with ELLA stent and followed for a median of 1302 days (IQR = 520–1641 days). Ten had no further treatments; 3 relapsed, one was lost to follow-up, and two underwent retransplantation and anastomosis redo. The median time to stricture recurrence was estimated with Kaplan–Meier analysis at 2915 days (Fig. 5A). Considering the event of clinical failure as stricture recurrence, the Log Rank analysis showed a statistically significant difference (*p* = 0.006) in case of recoil/residual stenosis (Fig. 5B). Previous treatments, stricture extension ≥ 10 mm, anastomotic vs intrahepatic involvement and development of post-procedural complications showed no statistical significance at univariate analysis.

Considering continuous variables like age at treatment, age at liver transplant, time from first PTC to ELLA placement, number of previous treatments, number of bilioplasty sessions before ELLA placement, bilioplasty balloon size, drainage catheter size and baseline direct bilirubin, ALP and GGT values, no significant differences were found between responders and non-responders (Table 3).

**Table 3:** Comparison of continuous variables between responders and non-responders (who developed stricture recurrence)

	Mann–Whitney U	Two-tailed sign. (*p*-value)
Age at treatment	879.0	0.652
Age at liver transplant	851.5	0.501
Time between the first percutaneous cholangiography and stent placement	879.0	0.652
Direct bilirubin at baseline	878.5	0.861
Alkaline phosphatase at baseline	792.0	0.683
Gamma-glutamyl transferase at baseline	848.5	0.762
Number of previous treatments	467.5	0.801
Drainage catheter size	616.0	0.709
Number of bilioplasty sessions	789.0	0.223
Bilioplasty balloon size	784.0	0.334

*sign.* significance

Regarding multivariate analysis, while at logistic regression analysis no factors were significantly correlated with stricture recurrence (Table 4), at the Cox proportional hazards model (Table 5) recoil/residual stenosis after bilioplasty was independently correlated with a shorter time to stricture recurrence (*p* = 0.005), with an HR of 5.334 (95% CI = 1.674–17). In the same model, the condition of having undergone previous treatments was protective to stricture recurrence (*p* = 0.033), with an HR of 0.256 (95% CI = 0.073–0.897). The other factors showed no significant correlation.

**Table 4:** Logistic regression with potential predictors of stricture recurrence

	Sign.
Etiology for liver transplantation	0.533
Previous treatments	0.902
Recoil/residual stenosis	0.319
Anastomotic vs intrahepatic	0.544
Stricture length (> 10 mm)	0.456
Complications	0.835

*Sign.* significance

**Table 5:** Cox proportional hazards model with potential predictors of time to stricture recurrence

	Sign.	Exp(B)	95.0% CI Exp(B)	95.0% CI Exp(B)
			Inferior	Superior
Previous treatments	0.033	0.256	0.073	0.897
Recoil/residual stenosis	0.005	5.334	1.674	17.000
Anastomotic vs intrahepatic	0.364	1.864	0.485	7.163
Stricture length (> 10 mm)	0.098	0.354	0.103	1.214
Complications	0.896	1.103	0.256	4.756

*Sign.* significance, *exp(B)* exponentiated value of the logistic regression coefficient (B), *CI* confidence interval

## Discussion

Biliary strictures remain the Achilles’ heel of pLT, occurring in 4–12% of children within 2 years after liver transplantation [22]. Compared with a 91% 10-year graft survival in pLT, the 10-year graft survival is only 73% in those with biliary complications [23, 24].

Our study found that pLT patients, affected by post-transplant biliary strictures and treated with self-expanding biodegradable stents, may have excellent outcomes without any significant safety issues. Freedom from stricture recurrence was achieved in 76% of patients at all five centers, in spite of different protocols. These results are comparable with conventional therapy, where literature reports a 70% success rate for conventional treatment with angioplasty and biliary drainage [14]. Our data is notable because the study population included a lot of patients who previously failed conventional therapy. Actually, biodegradable stents were mostly employed in case of recalcitrant biliary strictures before palliative metal stenting, surgical anastomosis redo or liver retransplantation. Biodegradable stenting offers a real and sustained treatment option in this complex patient group. Biodegradable stents were used in only 102 out of 326 pLT patients undergoing PTC across five institutions during the study period. Based on these excellent results, we anticipate that equivalent results could be achieved primarily in high-risk groups.

Similar findings with the self-expanding polydioxanone stent have already been shown in several adult studies [16–19, 25], while pediatric series are scarce [20]. One of the first and largest reports on biodegradable biliary stents was the multicenter study by Mauri et al [16], which showed that self-expanding polydioxanone stents provided good mid-term outcomes in patients with benign biliary strictures refractory to standard bilioplasty, without serious complications. Similar findings were observed in the large Spanish registry by De Gregorio et al [26], who observed only 26.6% of biliary stricture relapse after treatment with the ELLA device, without major adverse events. Another study involving 18 adults after liver transplant by Battistel et al [19] showed a 72% success rate with only one complication. An interesting study including adult and pLT patients treated with the same type of biodegradable stent was published by Dopazo et al [26], who also observed a clinical success rate of 75% without major complications.

While the above-mentioned studies mainly involved patients with recalcitrant biliary strictures, a recent publication by Quintero et al [20] reported improved outcomes in 43 pLT patients with success rates even above 90% in the mid-term when treatment anticipation was performed with primary stenting. The expected advantage of treatment anticipation is twofold: to enhance the efficacy of treatment and to improve the tolerability of treatment, avoiding the internal-external drainage catheter. Notably, in our study, 18 patients received the biodegradable stent during the first bilioplasty session, without maintenance of an internal-external biliary drainage. This represents the greatest innovation provided by biodegradable stents, which have the potential to improve the management of biliary strictures. Early placement of biodegradable stents that spare internal-external drainage catheters may reduce the number and duration of hospital stays for children, which greatly impacts their quality of life. This is also supported by our findings, where the number of previous treatments, the number of bilioplasty sessions and the indwelling time of the internal-external drainage catheter, compared to primary stenting, were not associated with a significant impact on treatment efficacy. However, the earlier adoption of such an approach was limited by the lack of CE mark and FDA approval during most of the study period. Nevertheless, the ELLA device has now received the CE mark, and it is largely available on the market in Europe.

We reported the largest multicenter series of biodegradable stents implanted in pLT patients. Despite the retrospective design, the study provided detailed information about stricture and procedure characteristics. The etiology of the biliary stricture was unknown in the majority of cases compared to the few ones in which it was supposed to be related to arterial obstruction, a condition known to result in ischemic cholangiopathy-related graft failure. Different from published series, which report outcomes of variable percutaneous treatments [9–12], our research included only patients treated with biodegradable stents.

We did not find a correlation between the tested variables and the development of stricture recurrence. However, we identified factors that were independently associated with a shorter time to recurrence, namely the presence of recoil/residual stenosis after bilioplasty. In contrast, previous treatments resulted in a longer time to progression. These findings provide evidence of the complexity and the multifactorial nature of biliary complications after pLT, with limited availability of reliable prognostic criteria.

Alternatives to biodegradable stents exist and are represented by removable plastic stents and metal stents. The former are not routinely used by interventional radiologists due to the invasiveness of retrieval procedures. The latter, although convenient, are susceptible to blockage due to accumulation of bile sludge or tissue overgrowth and the stent retrieval procedure may cause damage to the bile duct [27, 28]. Covered metal stents are not suitable for this clinical application since they would obstruct the branch ducts of the small split liver grafts.

Although the mechanical properties of polydioxanone are poor and its Young’s modulus is relatively low, published results are encouraging. Animal studies have shown that partial disintegration of the stent occurs in 8 weeks, with complete clearance between 13 and 20 weeks after implantation, without requiring removal [29]. While there have been concerns about the induction of inflammation or granulation tissue formation in other biodegradable stent applications (e.g., esophagus), such findings have been infrequently reported in the biliary tract of animals [29, 30], indicating a favorable safety profile for this device. Other compounds, like poly-L-lactic acid-based polymers, have been introduced with commercially available devices such as the Archimedes stent (Qualimed). Even though this compound is highly biocompatible, it lacks elasticity and the degradation by hydrolysis is poorly predicted. A preliminary study [31] reported the use of Archimedes in pLT patients, but new data on its percutaneous use are lacking, and its appropriate application seems to be the prophylactic placement at the time of surgery [32]. Magnesium-based alloys, which were originally tested in the coronary circulation [33], have also been recently introduced in commercially available stents like the Unity-B (Qualimed) device, which has theoretical advantages compared to the ELLA stent: a lower profile of deployment; balloon expansion for a more precise placement; and higher radial force. However, it is currently available only the fast degradation profile that dissolves in a variable time ranging from 1 to 3 months, which appears too short to provide long-term efficacy.

Although this multicenter study included a substantial number of patients, the absence of standardized practice among centers prevents us from establishing an optimal protocol for managing biliary strictures after pLT. Even within the adult population, where there is a larger body of literature, standardized protocols are lacking due to the previous absence of CE and FDA clearance. This investigation, which has yielded promising data on safety and efficacy, may act as a basis for future prospective trials to determine the best timing for PTC and stenting. The goal would be to reduce patient discomfort associated with internal-external drainage catheters and multiple procedures. Now that the ELLA stent has received the CE-mark, it is the authors’ opinion that, though not 100% effective in the long-term, biodegradable stents could be increasingly used to reduce the invasiveness of percutaneous procedures, leading to fewer admissions, less anesthesia, shorter hospital stays, and improved quality of life for pediatric patients.

This study has limitations: the retrospective design prevented us to consider other potentially interesting variables such as the time from transplant to first stricture detection; the lack of a standardized protocol among centers and the off-label use of the stents which may have biased their availability; the lack of a homogeneous follow-up: some of the pLT recipients live abroad from the enrolling centers, and are followed routinely by their local facilities. These limitations may be overcome in prospective clinical trials and multicenter registries.

## Conclusions

Self-expanding biodegradable biliary stents made of polydioxanone appeared to be safe and effective for the treatment of benign biliary stricture after pLT in a multicenter series of 102 patients. Some factors may predict worse outcomes and should be considered for appropriate patient selection and therapy individualization.

## Abbreviations

ALP: Alkaline phosphatase
GGT: Gamma-glutamyl transferase
HR: Hazard ratio
IQR: Interquartile range
OR: Odds ratio
pLT: Pediatric liver transplantation
PTC: Percutaneous transhepatic cholangiography
